# Exercise-induced motor improvement after complete spinal cord transection and its relation to expression of brain-derived neurotrophic factor and presynaptic markers

**DOI:** 10.1186/1471-2202-10-144

**Published:** 2009-12-04

**Authors:** Matylda Macias, Dorota Nowicka, Artur Czupryn, Dorota Sulejczak, Małgorzata Skup, Jolanta Skangiel-Kramska, Julita Czarkowska-Bauch

**Affiliations:** 1 Department of Neurophysiology, Nencki Institute of Experimental Biology, (3 Pasteur Str), Warsaw (02-093), Poland; 2 Department of Molecular Neurobiology, Nencki Institute of Experimental Biology, (3 Pasteur Str), Warsaw (02-093), Poland; 3 Department of Experimental Pharmacology, Medical, Research Centre, Polish Academy of Sciences, (5 Pawińskiego Str), Warsaw, (02-106), Poland; 4 Institute of Molecular and Cellular Biology, (4 Trojdena Str), Warsaw, (02-109), Poland

## Abstract

**Background:**

It has been postulated that exercise-induced activation of brain-derived neurotrophic factor (BDNF) may account for improvement of stepping ability in animals after complete spinal cord transection. As we have shown previously, treadmill locomotor exercise leads to up-regulation of BDNF protein and mRNA in the entire neuronal network of intact spinal cord. The questions arise: (i) how the treadmill locomotor training, supplemented with tail stimulation, affects the expression of molecular correlates of synaptic plasticity in spinal rats, and (ii) if a response is related to BDNF protein level and distribution.

We investigated the effect of training in rats spinalized at low thoracic segments on the level and distribution of BDNF immunoreactivity (IR) in ventral quadrants of the lumbar segments, in conjunction with markers of presynaptic terminals, synaptophysin and synaptic zinc.

**Results:**

Training improved hindlimb stepping in spinal animals evaluated with modified Basso-Beattie-Bresnahan scale. Grades of spinal trained animals ranged between 5 and 11, whereas those of spinal were between 2 and 4. Functional improvement was associated with changes in presynaptic markers and BDNF distribution. Six weeks after transection, synaptophysin IR was reduced by 18% around the large neurons of lamina IX and training elevated its expression by over 30%. The level of synaptic zinc staining in the ventral horn was unaltered, whereas in ventral funiculi it was decreased by 26% postlesion and tended to normalize after the training. Overall BDNF IR levels in the ventral horn, which were higher by 22% postlesion, were unchanged after the training. However, training modified distribution of BDNF in the processes with its predominance in the longer and thicker ones. It also caused selective up-regulation of BDNF in two classes of cells (soma ranging between 100-400 μm^2 ^and over 1000 μm^2^) of the ventrolateral and laterodorsal motor nuclei.

**Conclusion:**

Our results show that it is not BDNF deficit that determines lack of functional improvement in spinal animals. They indicate selectivity of up-regulation of BDNF in distinct subpopulations of cells in the motor nuclei which leads to changes of innervation targeting motoneurons, tuned up by locomotor activity as indicated by a region-specific increase of presynaptic markers.

## Background

The improvement of stepping ability in animals owing to locomotor training after complete spinal cord transection is well documented [1,2]. The involvement of neurotrophins in this process, particularly that of BDNF, has been postulated, as BDNF is crucial for activation and progress of recovery phenomena [3,4] and its synthesis depends on neuronal activity [5]. We have shown previously that locomotor exercise leads to the up-regulation of BDNF mRNA and protein expression in the intact spinal cord, particularly in the ventral horn [6-8]. Moreover, BDNF was found to modulate dendritic structure and spine formation [9,10] and to stimulate axonal branching [11]. All these observations suggest that up-regulation of BDNF caused by locomotor training could be a potent tool for remodeling of the spinal neuronal network in segments caudal to the lesion.

Experiments testing the regulation of BDNF signaling in the injured spinal cord did not bring consistent results. Widenfalk and co-workers [12] showed that six weeks after contusion of the spinal cord, the BDNF mRNA level, measured in segments caudal to the site of injury, was similar to that of intact rat. However, one month after contusion of the spinal cord [13] or its hemisection [14], BDNF mRNA expression was shown to be decreased in segments caudal to the injury. On the other hand, increased levels of BDNF were reported one and six weeks following complete spinal cord transection at low thoracic segments [15].

The data on the effect of physical exercise on the BDNF level in the spinal cord following injury are scarce and equivocal. Intensive locomotor training up-regulated BDNF mRNA above the control level after spinal cord hemisection [14], whereas moderate, voluntary, physical exercise did not have such an effect in the contused spinal cord [13].

More recently, it has been documented that transplantation of fibroblasts modified to produce neurotrophins (BDNF and NT-3) after complete transection of the spinal cord improved locomotor functions of the cat equally well as the locomotor training [16]. Therefore, the assumption of our study was that, if motor improvement after complete spinal cord transection depends on BDNF, and if postlesion BDNF availability is a limiting factor, then exercise should lead to an up-regulation of BDNF protein level, causing reorganization of the spinal neuronal network in segments caudal to the injury.

To examine our hypothesis we applied five-week locomotor training, starting one week after surgery. We evaluated BDNF levels concomitantly with synaptophysin expression as the latter protein is a presynaptic marker used to examine changes of synaptic connectivity [17-19]. Moreover, the distribution of synaptic zinc, frequently used to trace axonal sprouting, was examined [20-23]. Advanced image analysis was used to reveal an overall and region-specific distribution of markers.

Verifying whether the locomotor training in animals following complete spinal cord transection leads to upregulation of BDNF protein level in neuronal nets, as it does in intact animals, and whether these changes are accompanied by reorganization of the spinal network, is of particular clinical importance as it should help establish new therapeutic strategies.

## Methods

### Animals

Thirty two young adult, male Wistar rats, weighing 360-540 g at the end of the experiment, were used in the work described here. The animals were bred in the animal house of the Nencki Institute, Warsaw, Poland. They were given free access to water and pellet food and were housed under standard humidity and temperature at 12 h light/dark cycle. Procedures involving animals and their care were conducted in conformity with the institutional guidelines of the First Local Ethics Committee in Warsaw, which are in compliance with the European Community Council Directive (86/609/EEC). Three groups of animals were tested: intact control, spinal, and spinal subjected to locomotor training.

### Spinal transection and postoperative care

Twenty rats were anesthetized with Equithesin (0.4 ml/100 g b.w.) and their backs were shaved and disinfected with iodine at the incision sites. Skin and muscles were cut over the caudal thoracic segments with a fine scalpel. The position of the vertebrae was fixed by insertion of hooks into the connective tissue and muscles around the incision. A laminectomy was performed at the thoracic (T9/10) vertebrae. The dura was opened and Lidocaine (2% xylocaine) was applied on the surface of the cord. The spinal cord was then completely transected using surgical scissors and the gap between the rostral and caudal ends was enlarged by aspiration up to about 0.5 mm, washed with warm (around 36°C) 0.9% NaCl, and dried with absorbable cellulose. After careful inspection of the lesion area, the surrounding tissues were subsequently closed with surgical sutures; finally, the skin over the wound was closed with sterile stainless steel staples. About 6 ml of 0.9% NaCl was injected subcutaneously after the surgery. Enrofloxacin (Baytril 2.5%; 0.2 ml/kg) was administered subcutaneously at the end of the surgery and during 5 consecutive days in order to prevent infection. An analgesic Tolfedine (3 mg/kg, s.c.) was given during 3 postoperative days.

Immediately after surgery, the rats were placed in warm cages, covered with blankets, and inspected until they regained consciousness. They were returned to individual cages with full access to food and water as soon as they recovered after anesthesia. The animals were attended for general inspection three times daily during the first postoperative week and twice daily in the subsequent weeks, including cleaning of their bodies and manual bladder expression, if necessary. The animals had no significant health problems for weeks after spinalization, except for occasional bladder bleeding during initial post surgery days. Spontaneous micturition usually returned in the second week after surgery.

### Behavioral training

During a week preceding the experiment all tested rats were accustomed, twice daily, to walking on a motor driven treadmill belt at a speed of 0.05 m/s for 5-minute periods. After getting accustomed to the treadmill locomotion, 20 animals were spinalized at the T9/10 level. After one week recovery period, 10 spinal rats were left with no exercise except for one day of testing their motor abilities during the fifth week after the surgery. The other 10 rats were then accustomed to the treadmill walking with the forelimbs and rostral trunk placed on a platform located 1 cm above the belt, while the hindlimbs were placed on the running treadmill. The experimenter secured proper position of the trunk on the platform holding the animal's body and optimized the positioning of the hindlimbs for weight support by holding and manually pressing the proximal part of the tail. When the spinal animals became accustomed to the procedure, for the next 4 weeks the locomotor training was carried out five days a week, at a speed of treadmill belt between 0.05 - 0.1 m/s. The daily training consisted of four to six walking sessions, lasting about 4 min each, separated by about 30 min rest in the home cages. The animals were rewarded with their preferred food (corn flakes) after each session. All other animals (intact and spinal non-trained) accompanied the trained rats during their daily training in the experimental room, where they were kept in cages, handled, and rewarded occasionally with corn flakes. The control group consisted initially of 12 intact rats that got accustomed to the treadmill but were never trained, although one animal was excluded from further analysis due to bad tissue preservation.

The number of sequences of steps was counted by an experimenter. At least two consecutive steps performed alternatively on both hindlimbs were classified as a sequence and taken into account for further analysis. A side view of each rat walking on the treadmill was recorded after 2^nd ^and 3^rd ^week of training using a Panasonic VHS 5100 video camera at 30 frames per second.

### Materials

The primary polyclonal antibody against BDNF (N-20; sc-546) and the respective control peptide (N20P 546) were purchased from Santa Cruz Biotechnology, Inc. (Santa Cruz, CA, USA). Thanks to Dr. David Kaplan (HSC, University of Toronto), we also used an antibody against BDNF produced in his laboratory. Monoclonal anti-synaptophysin (MAB5258-50UG) was from Chemicon, and monoclonal anti-MAP-2 (M4403, clone HM-2, ascites fluid) was from Sigma. Other immunoreagents including Vector M.O.M. Kit for monoclonal antibodies, fluorescein conjugate with avidin DCS used for the amplification of fluorescent signal, standard and Elite Vectastain ABC detection kits, and secondary anti-rabbit antibody conjugated with Texas Red were all purchased from Vector Laboratories (Burlingame, CA, USA). Secondary antibodies conjugated with AlexaFluor were from Molecular Probes. All other chemicals and reagents were from Sigma, except for PFA (Merck, Germany), DPX (Park, UK), alcohols, and xylene (POCh, Poland).

### Immunohistochemistry

#### Tissue processing

Eighteen rats were subjected to immunohistochemistry (6 intact, 6 spinal non-trained, and 6 spinal and trained). The rats were anesthetized with lethal doses of sodium pentobarbital (80 mg/kg, i.p.) and perfused for 2-3 min via the ascending aorta with 200 ml 0.1 M phosphate-buffered saline (PBS), pH 7.4, and, subsequently for the next 20 min, with 400 - 500 ml of ice-cold fixative (2% paraformaldehyde plus 0.2% parabenzoquinone in 0.1 M PB). Spinal cords were removed from the vertebral columns and were postfixed in the fixative for 1.5 h at RT. The tissue was then cryoprotected overnight in 10% sucrose in 0.1 M PB at 4°C followed by 20% and 30% sucrose, until the tissue sank. The lumbar segments of the spinal cord were frozen with pre-cooled heptane (temp. around -30°C), placed on tissue holders, surrounded by the Jung tissue-freezing medium (Leica), and sectioned with a cryostat. Forty-micrometer transverse sections were collected free-floating in PBS, pH 7.4, to perform single-immunolabeling and complementary cresyl violet staining. Consecutive sections were collected to neighboring wells to assure that patterns of BDNF and synaptophysin labeling were analyzed on adjacent tissue areas. Five to six 40-μm sections per rat, representing L3 and L4 segments, were taken for analysis. For double-labeling studies, 16-μm glass mounted (BDNF/synaptophysin; BDNF/MAP-2) and 40-μm free-floating (BDNF/MAP-2) transverse sections were collected. Glass mounted sections were frozen at -20°C, whereas free-floating sections were collected and kept in anti-freeze medium until used.

Within each experiment, immunohistochemical processing of tissue sections from all groups was carried out simultaneously. The conditions of all procedures (dilutions of reagents and antibodies, washings, incubation time and temperature, blocking of nonspecific staining, and reaction development regimen), were rigorously maintained throughout the assays and were identical for the sections from all tested groups.

#### BDNF immunostaining

Two polyclonal anti-BDNF antibodies (from Santa Cruz and Dr Kaplan's) were used throughout the experiment. Santa Cruz polyclonal anti-BDNF antibody was extensively characterized in our previous experiments [6,8]. To confirm the effects evaluated with the Santa Cruz antibody, we also used the antibody kindly provided by Dr D. Kaplan. Both antibodies recognized mature BDNF protein but were raised against two different peptides from the carboxyl terminus of BDNF. Prior to labeling, sections were washed in PBS with 0.2% Triton X-100, pH 7.4 (PBST), incubated in a solution of 0.3% H_2_O_2 _in water for 20 min to quench endogenous peroxidase activity, washed extensively in PBST and, finally, incubated with 3% normal goat serum (NGS) in PBST for 60 min to reduce non-specific staining. The sections were then incubated overnight at 4°C with anti-BDNF rabbit polyclonal antibody (Santa Cruz, 1:1000 or Kaplan's 1:3000, in PBST + 1% NGS). The sections were then rinsed in PBST prior to 1 h incubation at room temperature with the respective biotinylated secondary antibodies from the ABC kit. Subsequently, after extensive washings with PBST, sections were incubated for 1 h with AB complex containing avidin-HRP conjugate. The sections were then washed with PBST and the antigenic sites were revealed by treating with 0.05% DAB and 0.01% H_2_O_2_. The reaction was terminated by addition of PBST and by subsequent PBS washings. The sections were mounted on gelatin-subbed slides, dehydrated in ascending alcohol concentrations, cleared through xylene, and covered with DPX resin.

#### Synaptophysin immunostaining

Immunofluorescent staining was performed on free-floating sections. After three 5-min rinses of the sections in PBS, nonspecific binding was blocked by incubating sections for 1 h with M.O.M. Blocking Reagent from Vector M.O.M. Kit for monoclonal antibodies. The sections were then briefly washed 3 times in PBS. The following steps were performed strictly according to the Vector protocol. Briefly, the sections were pre-incubated with the M.O.M. Diluent for 5-min at room temperature. The excess of the Diluent was tapped off and monoclonal anti-synaptophysin antibody (Chemicon) diluted 1:1000 in the M.O.M. Diluent, was applied on sections. After 30-min incubation followed by three 2-min rinses in PBS, the sections were incubated with biotinylated secondary antibody for 10 min. After three further rinses, the sections were incubated for 20 min with avidin DCS-fluorescein conjugate (Vector, 1:25). The sections were then washed three times in PBS and mounted onto glass slides, dried, and mounted with the Vectashield Mounting Medium for fluorescence.

#### Double immunolabeling of synaptophysin and BDNF

The sections were elaborated for synaptophysin, strictly as for a single immunolabeling, and after three 5-min rinses in PBS the sections were incubated overnight at 4°C with anti-BDNF rabbit polyclonal antibody (Santa Cruz, 1:1000). Next day, the sections were again washed 3 times (5 min each) in PBS and incubated with anti-rabbit secondary antibody conjugated with Texas Red (Vector, 1:200), washed three times (5 min each) in PBS, mounted onto glass slides, air-dried, and coverslipped with the Vectashield Mounting Medium.

#### Double immunolabeling of BDNF and MAP-2

The sections were washed in PBST and incubated with a solution mixture of 3% normal goat serum (NGS) and 3% normal horse serum (NHS) in PBST for 60 min, in order to reduce non-specific staining. The sections were incubated overnight at 4°C with anti-BDNF rabbit polyclonal antibody (Santa Cruz, 1:1000) combined with anti-MAP-2 mouse monoclonal antibody (Sigma AP-14 1:50), diluted with 1% NGS+1% NHS solution mixture in PBST. The sections were then rinsed in PBST prior to 1-hour incubation at room temperature with the respective secondary antibodies linked to Alexa Fluor 488 (green fluorescence dye, for BDNF labeling; 1:200) or Alexa Fluor 594 (red fluorescence dye, for MAP-2 labeling; 1:200). After several washes, the sections were mounted onto glass slides, air-dried, and coverslipped with the Vectashield Mounting Medium.

#### Control of immunolabeling specificity

**Figure 1:**
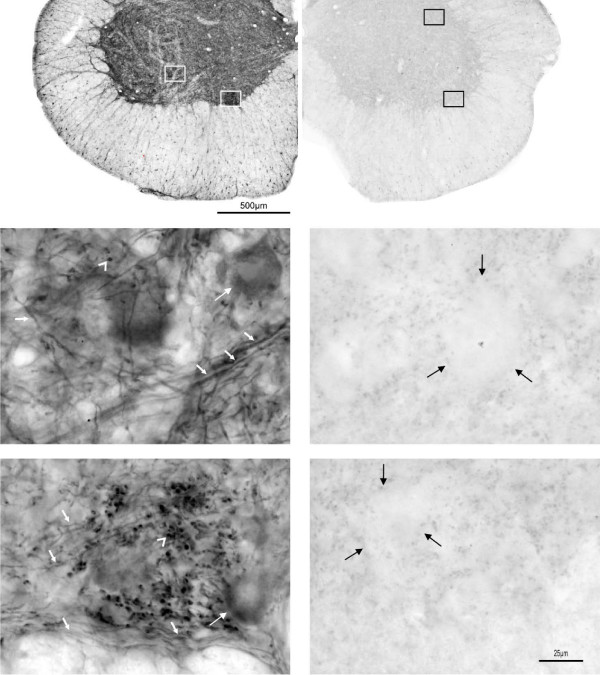
**Specificity of BDNF immunolabeling of spinal sections with the Santa Cruz anti-BDNF antibody**. As shown in control images (right) the labeling of perikarya and fibers, carried out with the anti-BDNF antibody (Ab) preadsorbed with control peptide, has been completely abolished. A weak, diffused signal seen in neuropil of the section incubated with the preadsorbed Ab and shown at higher magnification in the lower panel (right) was made visible owing to prolonged exposure time. However, cell bodies in lamina IX remain unstained and can be distinguished as white empty areas indicated by black arrows on right panel. When the photographs were taken under the same exposure conditions for the sections labeled with anti-BDNF Ab and with anti-BDNF Ab preabsorbed with a control peptide, the latter signal could not be seen. Scale bars, 500 μm in the upper panels and 25 μm in lower panels.

We performed a series of controls to validate the specificity of immunohistochemical profiles observed in our study. 1) Immunolabeling specificity was routinely examined by omitting a primary antibody in the incubation mixture. Under these conditions, no immunolabeling was ever detected. 2) In the initial series of experiments control immunostaining with the Santa Cruz antibodies preincubated with blocking peptides was performed (Figure 1). A non-specific staining was absent except for a faint background labeling, detected predominantly in the outermost part of the funiculi. 3) The specificity of staining in fluorescent labeling assays was verified in two ways, first by omission of the primary antibodies, and, second, by omission of the secondary antibodies. These tests resulted in lack of fluorescent staining. In double immunolabeling approach the labeling was controlled for each antibody separately, by omission of the secondary antibody while all other steps remained unchanged. These tests proved that none of the staining obtained in our experiments was due to non-specific fluorescence or filter bleed-through.

### Synaptic zinc histochemistry

#### Tissue preparation

Fourteen animals were used in order to visualize synaptic zinc. Since the quality of sections from the lumbar segments of one rat was not satisfactory, this animal was excluded from the study, thus the final analysis was performed on five intact, four spinal non-trained, and four spinal trained rats.

The rats were injected intraperitoneally with 2% sodium selenite in deionized water (20 mg/kg). After 60 min, animals were deeply anesthetized with sodium pentobarbital (100 mg/kg, i.p.) and injected into the heart with 0.2 unit of heparin (in 0.2 ml PB). The rats were perfused transcardially with 100 ml of ice-cold 0.1 M PB, at a flow rate of 20 ml/min, followed by 200 ml of ice-cold 4% paraformaldehyde in 0.1 M PB. After perfusion, spinal cords were removed from the vertebral column and cut onto segments which were postfixed for 4 h in 4% paraformaldehyde and then cryoprotected in 30% sucrose. The lumbar segments of the spinal cord were embedded in tissue-freezing medium (Leica) in separate molds to preserve their shape, frozen by immersion in cold heptane (-70°C), and stored at -70°C. Transverse sections (20 μm) were obtained on a cryostat, mounted onto gelatine/chrome-alum-coated glass slides, air-dried, and stored at -70°C until histochemical staining.

#### Synaptic zinc visualization

Spinal cord sections from the lumbar segments obtained from the three groups of rats described above were processed for histochemical staining simultaneously to avoid possible variations in reaction conditions. The histochemical development was performed according to Danscher [24], with slight modification by Czupryn and Skangiel-Kramska [25]. The frozen sections mounted on glass slides were allowed to dry at room temperature and then were progressively rehydrated with descending alcohol solutions (96% ethanol for 15 min, followed by 70% ethanol for 2 min and by 50% ethanol for 2 min), dipped in water, and finally in 0.5% gelatin solution. The slides were then immersed in freshly-prepared developing solution containing 37 mM silver lactate, 0.5 M hydroquinone and 40% arabic gum in 2 M citrate buffer (pH 3.5), and were incubated in the dark for 40 min at 26°C. We assessed the reaction time at this temperature in our preliminary experiments in order to optimize staining intensity. After washing for 20 min in 37°C running tap water, the sections were rinsed twice in deionized water, immersed for 12 min in 5% thiosulfate solution, and then rinsed again in de-ionized water. Finally, the sections were postfixed for 60 min in 70% ethanol, dehydrated in ascending series of alcohols, and coverslipped with Permount (Fisher Scientific).

### Sections analysis and quantification of results

The sections processed for different staining were examined using a Nikon Eclipse 80i microscope equipped with a monochromatic CCD camera Evolution VF (Media Cybernetics, Inc., Silver Spring, MD, USA). Image-Pro Plus 5.0 (Media Cybernetics, Inc., Silver Spring, MD, USA) digitizer and software and Neurolucida (MicroBrightField, Inc., Williston, VT, USA) were used for data analysis. The light source was stabilized during image acquisition to maintain the same illumination level at each imaging session, and the settings of the camera and the lamp were constant [26]. For bright field microscopy, shading correction was applied. Brightness and contrast were adjusted to obtain images as close as possible to those observed directly under the microscope. Figures were assembled using Adobe Photoshop software.

#### Analysis of BDNF labeling

**Figure 2:**
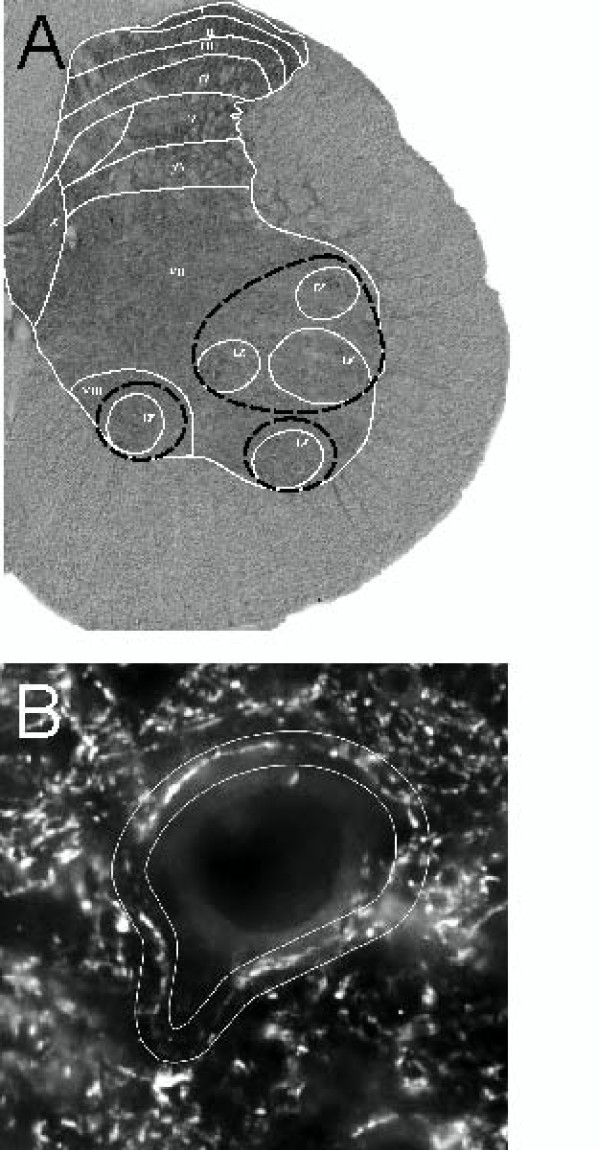
**Images of the spinal cord tissue sections with delineated areas which were subject to quantitative analysis**. A. Brightfield photomicrograph showing immunocytochemical labeling of BDNF on a cross section of the lumbar (L3/L4) segment of the spinal cord in the intact rat. Spinal cord laminae distinguished in L4 segment [27] are overlaid. Areas covering motor nuclei (lamina IX) delineated with dashed black lines were taken for quantitative evaluation of BDNF immunoreactivity. B. Photomicrograph showing immunofluorescence of synaptophysin around a large neuron in the ventral horn of the lumbar (L3/L4) segment of the spinal cord. Delineated areas (white lines) surrounding large neurons of lamina IX were taken for quantitative estimation of the synaptophysin immunofluorescence intensity.

Microscopic images for measurement of BDNF by densitometry were captured during one session, to ensure the same illumination level. At least three sections representing L3 and L4 segments from each rat were chosen for morphometry and densitometry of BDNF immunoreactive profiles of the perikarya, processes, and fibers. Profiles of the motor nuclei of lamina IX were outlined manually according to the rat brain atlas [27] and the densitometric analysis was performed within these outlines (Figure 2A). Only cell bodies with sharp, well-defined edges were taken into account, outlined, and evaluated for each section. The densitometric signal of the background was collected in the area occupied by corticospinal tract fibers and was subtracted from the BDNF signal of the cell bodies in every section. The images collected for densitometric analysis by means of Image-Pro Plus 5.0 were captured at a single focus plane.

Spinal sections were processed with two anti-BDNF antibodies, obtained from Santa Cruz (SC) and from Dr Kaplan. Both antibodies effectively labeled perikarya and processes. However, labeling with Kaplan's antibody produced punctuate labeling which marked perikaryonal edges better than SC antibody. Therefore, it was the antibody of choice for detailed analysis of BDNF IR in the perikarya. The SC antibody strongly labeled the dense network of processes and fibers, enabling detailed analysis of the fiber network.

For fiber network analysis, a skeletonization method using Image-Pro Plus 5.0 software was applied. After thresholding, extracted BDNF-immunopositive processes and objects were smoothed and subjected to "thinning filtering" procedure, which reduced the image to the skeleton. The skeletonized image was than compared to the original one to confirm whether this transformation did not bring substantial artifacts. This analysis allowed measuring (i) the density of extracted BDNF IR processes and fibers, (ii) their length, and (iii) the area occupied by BDNF IR processes and fibers in the tested area. This procedure was sensitive for extracting all strongly-labeled objects (without the cell bodies, which were subtracted in this analysis). The boundaries of the spinal laminae were verified microscopically at Nissl stained cross-sections.

To confirm the results obtained with skeletonization technique, we tracked BDNF IR processes and fibers with Neurolucida 7 software. This analysis was performed in part of the ventro-lateral motor nucleus. An area of about 50 000 μm^2 ^was delineated and all clearly visible BDNF IR processes were tracked along the Z-axis at multiple focal planes. One representative section from the L4 segment per rat was taken for the analysis. Changes in diameter of tracked processes were also taken into account. This analysis allowed measuring (i) the density of BDNF IR processes, (ii) their length, and (iii) volume.

#### Analysis of synaptophysin immunofluorescence

All images were captured at identical exposure times during one microscopic session in order to ensure the same illumination level. Three sections per rat, separated from each other by at least 240 μm, were chosen for analysis. Analysis of synaptophysin immunofluorescence (IF) was confined to large neurons within lamina IX of the L3-L4 segments of the spinal cord as only their cell bodies had sharp, well-defined edges and visible nuclei. The mean numbers of large neurons per rat selected for further analysis were: 28 in the intact, 33 in spinal, and 25 in spinal trained animals. The Image-Pro Plus software was used to encircle the perimeter of each neuron manually (Figure 2B). Mean optical density within the outlined areas of 4 μm in width was measured. Averaged optical density of neuronal perimeters was calculated for each animal and these results were used for statistical analysis. Additionally, digital images were captured by a confocal inverted microscope Leica DM IRE2 with optical slice of 1 μm, using a HCX PL APO 63× oil-immersion objective lens.

#### Analysis of double immunofluorescence

Digital images were captured by a confocal inverted microscope (Leica DM IRE2) with optical slice of 0.5 μm, using a HCX PL APO 63× oil-immersion objective lens. To detect and evaluate the contacts and overlap of structures labeled with BDNF/synaptophysin and BDNF/MAP2, optical slices of 0.5 -1.0 μm were digitally merged.

#### Analysis of synaptic zinc staining

Images of individual sections, stained for synaptic zinc within the ventral quadrants of the L3-L4 spinal segments, were examined under a microscope and the staining patterns were evaluated by two investigators. Typically, 3-4 sections per rat were subsequently acquired for quantitative analysis. Measurements of the relative optical density (R.O.D.) values representing gray level within sections in arbitrary units were performed after importing digital images from Image-Pro Plus 5.0 acquisition system into MCID M4 image analysis system (Imaging Research Inc., Saint Catherine, Ontario, Canada). Zinc staining levels were analyzed within the ventrolateral and ventral funiculi in four rectangular defined areas (7300 μm^2 ^each), and then R.O.D. values were averaged for each section. The area of the pyramidal tract, a region consistently showing low staining level, was taken as a reference structure, and the averaged reference R.O.D. values for each section were calculated. A ratio of R.O.D. values for each section was determined by calculating the mean value from the area of interest divided by values obtained from the reference area, and finally the averaged mean ratio for each rat was calculated.

### Statistical analysis

The Kruskall-Wallis analysis followed by the Dunn post-hoc test or one-way Anova followed by the Tukey-Kramer test were used for statistics. The Sign test was also applied to analyze the progress in the locomotor capability of the spinal trained rats. The level of significance was set at p < 0.05.

## Results

### The locomotion of rats after complete spinal cord transection and the effect of treadmill training on the motor abilities

In all spinal trained rats tested, there were generally no movements of the hindlimbs for the first 8-10 days after a complete section of the spinal cord at T9/10. The hindlimbs were dragged on the treadmill belt with the feet on their dorsum. Strong pressure stimulation of the tail caused initial short episodes of alternative locomotor-like movements at the hips and knees during second post-surgery week. The movements consisted mainly of occasional flexion at these joints, with the limbs being dragged on the belt surface. At that stage, neither active support of the hindlimbs nor plantar foot contacts were observed.

By the third week after spinalization (i.e., during the first week of the training) each rat spent about 20 min daily with its hindlimbs on the treadmill belt (at a speed between 0.05 - 0.1 m/s). The rats then began to place the plantar surface of their feet on the treadmill and showed some weight support during the stance phase. Hyper-adduction at the hips was regularly observed during treadmill walking in two rats, causing instability of their hindquarters. In the other three rats, we observed hyper-abduction at the hips. At that stage, the feet occasionally cleared the surface of the treadmill during the swing phase but only when the tail was stimulated.

From the fourth week after spinalization, we began to observe regular sequences of steps with occasional weight support during the stance phase. The number of steps performed on the plantar aspect of the feet increased gradually in the course of the training. However, until the end of locomotor training, the rats were often dragging their feet during the swing phases and only one hindlimb cleared the surface of the treadmill. In the stance phase, when some weight support was observed, spinal rats placed the plantar surface of the toes on the treadmill but they were unable to use the whole feet, as intact rats did.

**Figure 3:**
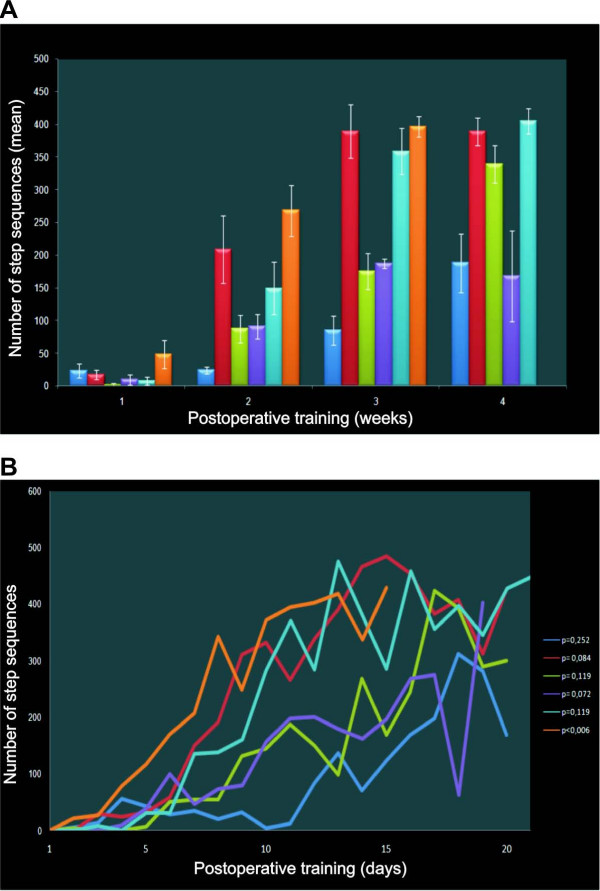
**The total number of sequences of hindlimb steps performed by spinal rats during 4 weeks of treadmill walking training starting 14 days after complete spinal cord transection**. At least two consecutive steps performed alternatively on both hindlimbs were classified as a sequence and taken into account for further analysis. The results obtained by six spinal trained rats are illustrated, except for the 4th week of training, when one animal was excluded from further training due to foot injury. A. Each bar represents the mean ± SEM obtained by a single animal in consecutive weeks of training. The progress in locomotor capability evaluated for weekly averaged data was significant (Sign test; p < 0.01). B. The number of step sequences of individual animals, analyzed day by day, increased but this non-monotonic progress was non-significant in all but one rat (the Sign test, p values are shown on the right side of the Figure).

This gradual improvement of locomotor ability during the treadmill training has been observed, to different degrees, in all trained animals. The number of step sequences increased with the training (Figure 3). However, all spinal rats, even those which showed clear improvement of their locomotor ability in the course of the treadmill training, remained paraplegic when left without stimulation of the tail on the treadmill or during spontaneous overground locomotion in an open-field situation. Thus, a combined treadmill exercise and pressure stimulation of the tail is a prerequisite for progress in the spinal stepping ability on both hindlimbs. To quantify the locomotor capability of spinal animals we applied Basso-Beattie-Bresnahan (BBB) scale [28], a modified version for the rats walking on both hindlimbs on the treadmill (while both forelimbs were placed on a platform) [29]. Spinal trained rats reached grades varying between 5 (at level 2) and 11 (at level 4) of modified BBB scale [29]. Practically, all trained animals showed occasional or frequent plantar foot placement although this improvement was not always symmetrical [29]. The three best- performing rats showed occasional weight-supported plantar steps with alternative movements (grade 11). Five weeks after spinal cord transection, non-trained rats usually performed irregular alternative movements of both hindlimbs with the feet dragged on the foot dorsum, reaching grades 2- 4 (at level 2) [29].

### The effect of training on synaptophysin expression in ventral quadrants of the lumbar segments of spinal rats

**Figure 4:**
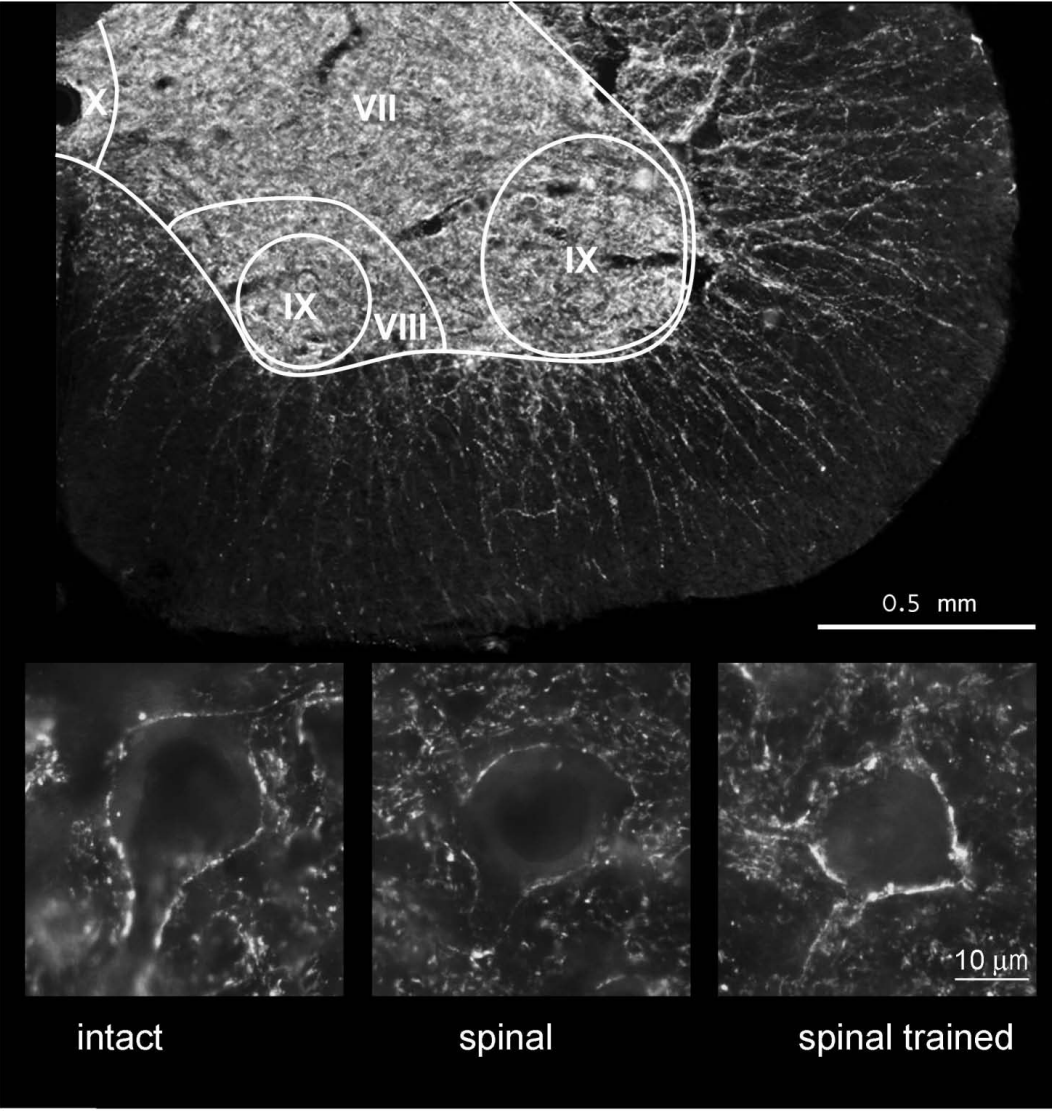
**Photomicrographs showing immunofluorescence of synaptophysin in the ventral horn of the lumbar (L3/L4) segment of the spinal cord**. Upper panel: a representative example of a cross section of a ventral quadrant of the lumbar segment in an intact rat stained for synaptophysin. Spinal cord laminae are distinguished in L3 segment according to The Rat Brain atlas [27]. Scale bar, 500 μm. Lower panel: representative photomicrographs showing synaptophysin immunofluorescence distribution around large neurons of lamina IX in the lumbar segments of the spinal cord of intact (left), spinal (middle) and spinal trained (right) animals, six weeks after complete spinal cord transection. Note the weaker immunofluorescence around the neuron in the spinal animal than in the intact and spinal trained rat. Scale bar, 10 μm.

Synaptophysin immunoreactivity was widely distributed in the neuropil of the spinal gray matter at the L3/4 segments of the intact rats (Figure 4). Synaptophysin signal accumulated in immunofluorescent deposits of different size. The immunofluorescent (IF) staining was also observed on bundles of fibers and processes spanning through the white matter.

The IF signal accumulated on perikarya and proximal dendrites of large neurons in lamina IX (Figure 4). This type of labeling did not allow differentiating of cell nuclei except for the largest cells in laminae IX, where a diffused, weak synaptophysin labeling in the cytoplasm allowed distinguishing between cytoplasm and nuclei, the latter being devoid of staining. Therefore, only the largest neurons in lamina IX were subjected to quantitative evaluation as their nuclei and borders of neuronal perikarya were clearly visible.

**Figure 5:**
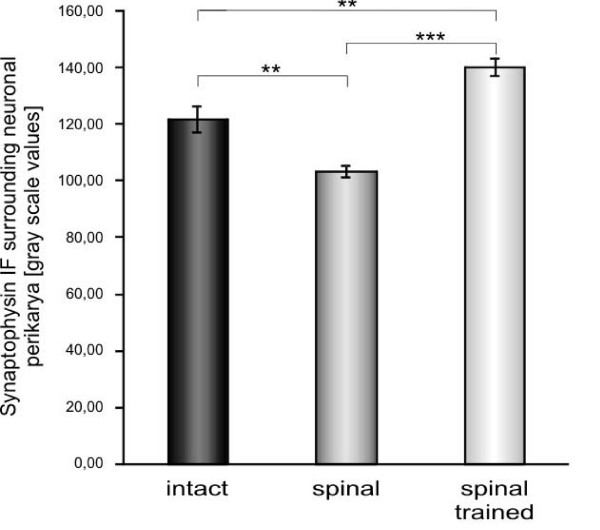
**The effect of spinalization and locomotor training on the intensity of synaptophysin immunofluorescence in the surrounding of perikarya of large neurons of lamina IX, in L3/L4 segments of the spinal cord**. Optical density of synaptophysin immunofluorescence (IF) around perikarya was measured as indicated in Figure 2B and expressed in gray scale levels as the group means (± SD). Three sections per rat from L3/L4 segments were chosen for analysis. The mean numbers of large neurons per rat, selected for further analysis, were 28, 33 and 25 in the intact (n = 6), spinal (n = 6), and spinal trained (n = 5) rats, respectively. Two and three asterisks correspond to p < 0.01 and p < 0.001, respectively (Tukey-Kramer post-hoc test).

It appeared that both the effect of a spinalization and locomotor training on the synaptophysin IF level were significant (one-way ANOVA, F_4,10 _= 26.9, p < 0.0001). Six weeks after complete spinal cord transection, the optical density of synaptophysin IR, calculated within manually outlined surrounding of the large neurons of lamina IX at the L3/L4 segments, was lower by over 18% than in the intact rats (Tukey-Kramer post-hoc test, p < 0.01) (Figures 4 and 5). Long-lasting treadmill locomotor training enhanced synaptophysin IR levels around the neuronal perikarya and proximal dendrites of large neurons of lamina IX by over 30% above those found in spinal non-trained animals (Tukey-Kramer post-hoc test, p < 0.001). This elevated level of synaptophysin IF was higher by about 12% compared to that found in the intact spinal cord (Tukey-Kramer post-hoc test, p < 0.01) and resulted in increased coverage of large neurons perikarya with synaptophysin-positive terminals (Figures 4 and 5).

### The effect of locomotor training on synaptic zinc distribution in ventral quadrants of the lumbar segments of spinal rats

**Figure 6:**
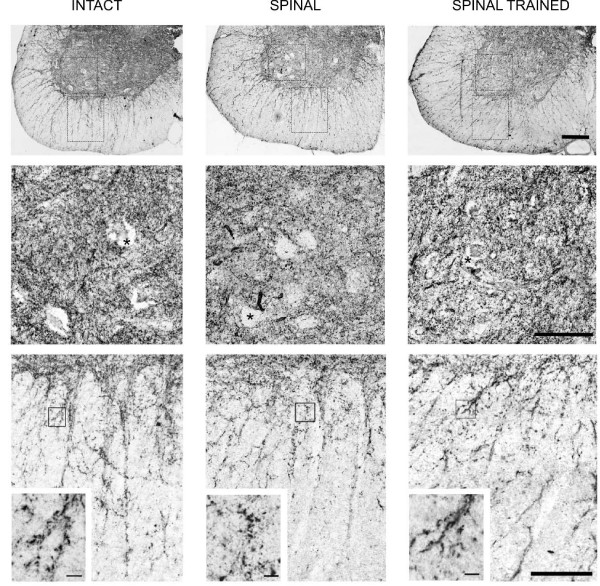
**Synaptic zinc staining pattern in the lumbar spinal cord of intact (left), spinal (middle), and spinal trained (right) rats**. Upper panel: representative photomicrographs of 20-μm-thick cross-sections of ventral quadrant of the lumbar segments of the spinal cord. The framed areas of lamina IX are shown enlarged in the middle row and those sampling ventral funiculus are shown in the bottom row. Asterisks (middle row) mark large cell bodies which are devoid of the synaptic zinc-positive grains. Note that in the gray matter there are no visible differences in the synaptic zinc distribution between intact, spinal and spinal trained rats. Insets in the bottom row show, at high magnification, the bundles of processes radiating from the gray into the white matter demarcated by zinc-positive grains. This grainy encrustation seemed to be more prominent on fibers in the intact than in two other groups and was assessed quantitatively (see Figure 7). Scale bars: upper row, 250 μm; middle and bottom rows, 100 μm, insets, 10 μm.

In the lumbar segments of the intact spinal cord, synaptic zinc staining revealed dark grains decorating a well-defined heterogeneous network of fibers (Figure 6). In the gray matter, the staining was more intense compared to the white matter. At high magnification, we were able to distinguish separate grains, which presumably represented single zinc-containing terminals and clusters of grains representing closely located zinc-ergic synapses. Zinc-positive grains clearly demarcated the surface of neuronal processes and fibers. Large cell bodies in lamina IX were devoid of such stain grains, and these cells were identified under the Nomarski phase contrast (not shown). The grains in the vicinity of perikarya of large neurons likely correspond to "zinc-ergic" terminals making synapses on them. Besides grains, we observed dispersed "blurry" zinc stain of neuropil which was almost evenly distributed within the gray and white matter, except for cell bodies, in which this type of zinc staining was almost never observed.

There were numerous bundles of processes radiating from the gray into the white matter demarcated by zinc-positive grains. They were visible especially well in the ventral and dorso-lateral funiculi (Figure 6).

**Figure 7:**
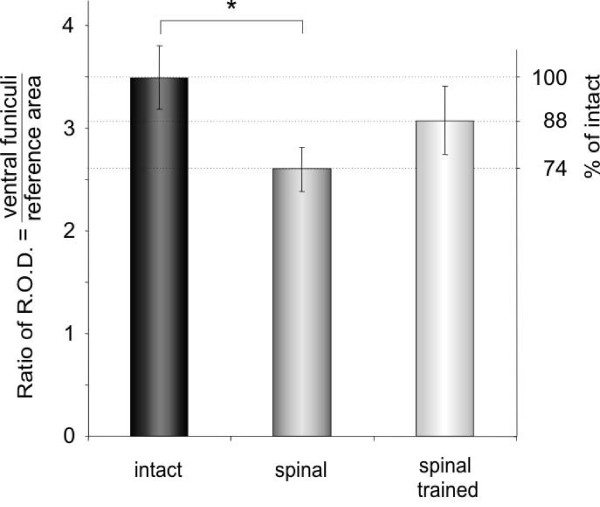
**A quantitative analysis of the effect of spinalization and locomotor training on the synaptic zinc staining density in the ventral funiculi of the lumbar (L3/L4) segments**. The data are presented as ratios of relative optical density (R.O.D.) of zinc staining in the ventral funiculus to R.O.D. in the pyramidal tract, which served as a reference area. Mean ± SD values obtained from the intact (n = 5), spinal (n = 4), and spinal trained (n = 4) rats are shown. Right axis shows a percentage change in relation to values obtained in intact rats. Statistically significant difference between groups is indicated by asterisk (p < 0.05, Dunn post hoc test).

Analysis of the staining pattern in spinal rats did not visually reveal detectable changes in the gray matter. However, a quantitative evaluation showed that zinc encrustation on fibers radiating from the gray into the white matter appeared changed in the spinal groups, both trained and non-trained. The Kruskal-Wallis test showed that an effect of spinalization on the zinc staining density was significant (p < 0.0191). Six weeks after transection the density of zinc-encrusted processes in the ventral funiculi was lower by 26% than in intact rats (Dunn post hoc test, p < 0.05; Figure 7). Interestingly, the locomotor training of spinal rats led to a tendency to normalization. This was shown by an 18% higher level of zinc-positive signal in trained than non-trained rats (Figure 7). Taken together, after locomotor training of spinal rats, we observed an improvement in the synaptic zinc distribution along processes radiating from the spinal gray to the white matter in segments below the injury site. We propose that the number of zinc-ergic synapses (demarcated by zinc-positive grains) on processes in the white matter increases with post-injury locomotor training.

### The effect of training on BDNF expression in ventral quadrants of the lumbar segments of spinal rats

**Figure 8:**
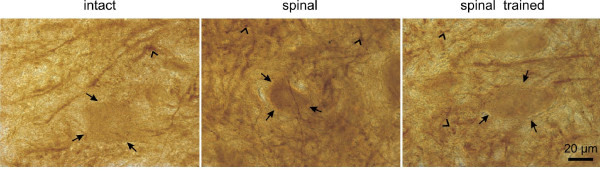
**Brightfield photomicrographs showing immunocytochemical labeling of BDNF on the cross-sections of the lumbar (L3/L4) spinal cord in lamina IX of the intact (left), spinal (middle) and spinal trained (right) rats**. The arrows point to BDNF IR perikarya of large neurons of lamina IX, the contour arrowheads point to BDNF immunopositive deposits. Scale bar, 20 μm.

In the intact spinal cord, BDNF IR localized predominantly in neuropil of the spinal gray matter, with the densest accumulation in lamina IX, in agreement with our earlier observations [6,8]. BDNF IR was detected in neuronal perikarya and in strongly immunopositive dense mesh of processes surrounding the large neurons in lamina IX (Figure 8). In addition, we have observed numerous BDNF IR bouton-like accumulations around large neurons of lamina IX. This pattern of BDNF IR was evident when the SC antibodies were used for labeling. The BDNF expression revealed with the Kaplan's antibody was localized both in the perikarya and in processes but the labeling of processes was rather faint compared to the perikaryonal staining.

**Figure 9:**
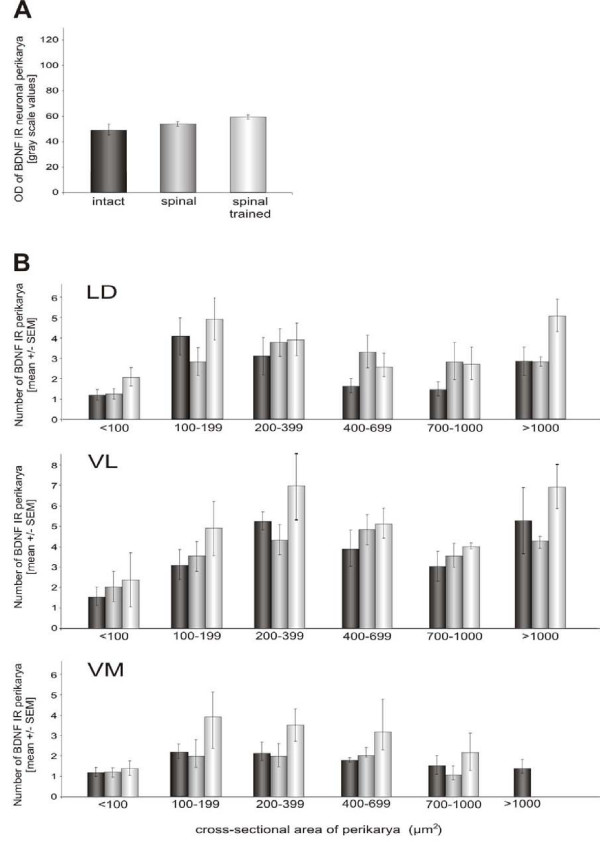
**A quantitative analysis of the effect of spinalization and locomotor training on BDNF immunoreactivity in the lumbar (L3/L4) segments of the spinal cord**. A. Staining intensity of BDNF immunoreactivity (IR) measured in neuronal perikarya of lamina IX labeled with Kaplan's antibody (mean ± SEM). Locomotor training of spinal animals led to 20% increase of perikaryonal expression of BDNF IR within the profiles of the motor nuclei. B. A comparison of BDNF IR in the perikarya of sized cells in the latero-dorsal (LD), ventro-lateral (VL), and ventro-medial (VM) motor nuclei. Note, that in the VL and LD nuclei, two subpopulations of BDNF IR cells, those ranging between 100 and 400 μm^2 ^and those above 1000 μm^2^, tended to be more numerous in spinal trained than in spinal or intact animals. Bars in A and B represent means ± SEM from five intact, five spinal and four spinal trained rats.

**Figure 10:**
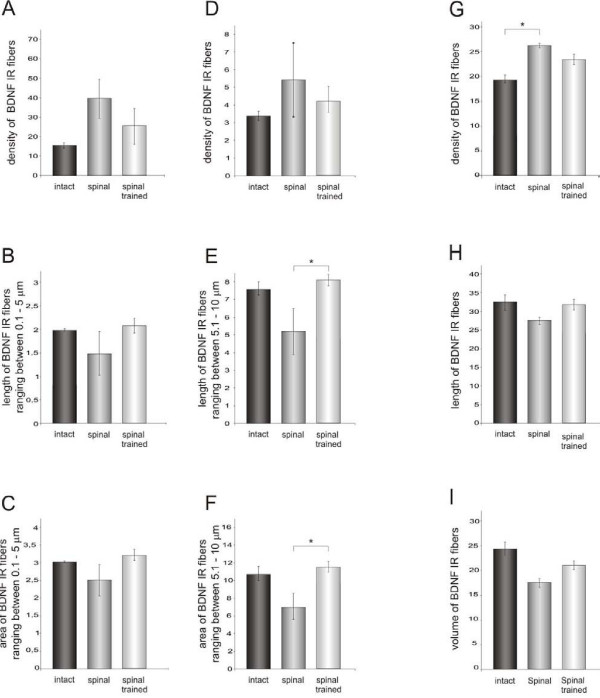
**BDNF immunoreactivity in processes and fibers of motor nuclei in the L3/L4 segments of the spinal cord of the intact, spinal and spinal trained rats labeled with the Santa Cruz antibody**. The left and central columns present data analyzed with a skeletonization technique (Image-Pro Plus software). Density (A), length (B) and area (C) occupied by BDNF immunoreactive (IR) processes and fibers ranging in length from 0.1 to 5 μm (mean ± SEM). Central column: density (D), length (E) and area (F) occupied by BDNF IR processes and fibers ranging in length from 5.1 to 10 μm (mean ± SEM). Six intact, five spinal and six spinal trained rats were used and at least three sections per animal were analyzed. The density of BDNF IR processes tended to be higher in the spinal than in intact animals and locomotor training tended to normalize it (A, D). The mean length of BDNF IR processes and the area occupied by them (B, E, C, F) were lower in the spinal than in the intact animals and training also normalized them (p < 0.02, one-way Kruskal-Wallis test). Asterisk corresponds to p < 0.05 (Dunn post-hoc test). Right column: density (G), length (H) and volume (I) of BDNF IR processes and fibers traced with the aid of Neurolucida software in the area of 50 000 μm^2 ^in the ventro-lateral nucleus. One section per animal was analyzed. Higher density of the BDNF IR network of processes and fibers observed in the spinal animals (G) was composed of shorter and thinner elements than in the intact and spinal trained animals (p < 0.05, one-way Kruskal-Wallis test). Asterisk corresponds to p < 0.05 (Dunn post-hoc test).

Six weeks after spinal cord transection, the overall BDNF IR levels in the ventral horn (evaluated on the sections labeled with the SC antibodies) were increased by 22%. That increase appeared to prevail in the fiber and processes compartment, as BDNF IR in neuronal perikarya was similar to that observed in the intact rats. A detailed analysis of changes in perikaryonal labeling is shown on Figure 9 whereas that in fiber labeling is presented on Figure 10.

Figure 9A shows that locomotor training of the spinal animals led to a 20% increase of perikaryonal expression of BDNF IR within the profiles of the motor nuclei, at the L3/L4 segments, compared to the intact rats. However, the statistical analysis did not reveal significant effect of spinal cord transection or training on the BDNF IR in the neuronal perikarya in the motor nuclei (one-way Kruskal-Wallis followed by Dunn post-hoc tests). Figure 9B shows an analysis of the response of BDNF IR cells to the training in three motor nuclei. In the biggest motor nucleus, located ventrolaterally (VL), two subpopulations of BDNF IR cells, those ranging between 100 and 400 μm^2 ^and those above 1000 μm^2^, tended to be more numerous in spinal trained than in spinal non-trained or intact animals. A similar distribution was found in the laterodorsal nucleus (LD) (Figure 9B). In the ventromedial (VM) motor nucleus, BDNF IR cells in all classes ranging between 100 and 900 μm^2 ^tended to be more numerous in the spinal trained than in intact and spinal non-trained animals. To summarize, this analysis suggests that not all classes of the ventral horn neurons (and glial cells) are equally responsive to the locomotor training.

Next, we assessed whether locomotor training of the spinal rats produces morphological changes in the network of BDNF IR processes in the ventral horn. BDNF immunopositive processes and fibers collected in one focal plane were analyzed by means of the skeletonization technique (see Method section). This analysis revealed that over 80% of BDNF IR processes were within the range of 0.1 and 5 μm of length, whereas about 16% ranged between 5.1 and 10 μm. Figure 10(A, D) shows that the density of BDNF IR processes tended to be higher in the spinal than in the intact animals and that locomotor training tended to normalize it. However, the mean length of BDNF IR processes and the area occupied by them were lower in the spinal than in the intact animals and training also normalized them (Figure 10B, E). In the subpopulation of longer BDNF IR processes (Figure 10E, F) both the effect of spinalization and training on their length was significant (p < 0.0151, one-way Kruskal-Wallis). These processes were significantly longer in the spinal trained than in spinal animals (p < 0.05, Dunn post-hoc test) (Figure 10E). Similarly, both the effect of spinalization and training on the area occupied by BDNF IR processes was significant (p < 0.021, one-way Kruskal-Wallis). The BDNF IR processes occupied bigger area in the spinal trained than in the spinal non-trained rats (p < 0.05, Dunn post-hoc test) (Figure 10F). These observations were consistent with those analyzed with Neurolucida software, meaning that the higher density of the BDNF IR network of processes and fibers observed in spinal animals was composed of shorter and thinner elements than in the intact and spinal trained animals (Figure 10G, H, I).

**Figure 11:**
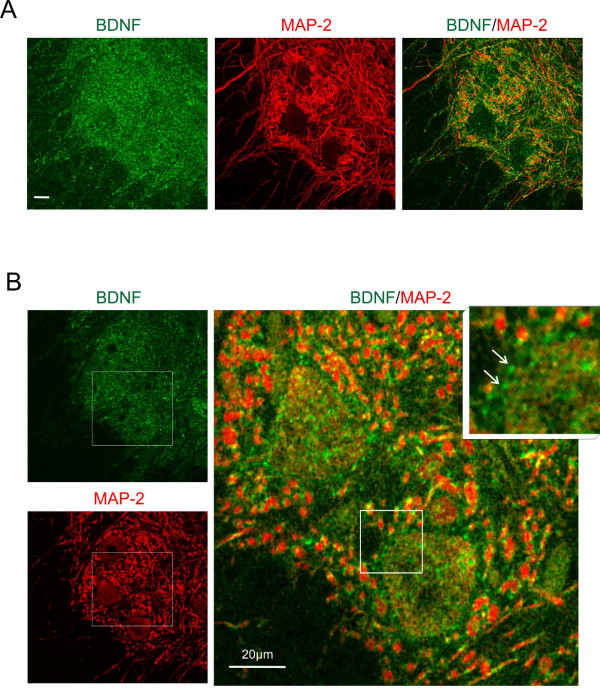
**Co-localization of BDNF (green) and dendritic MAP-2 (red) immunofluorescence in double stained cross-sections of the lumbar (L3/L4) segments in the intact spinal cord**. A. Confocal images show the appearance of BDNF immunopositive and dendritic MAP-2 immunopositive structures in the ventral horn. Merged images (right) show regions of presumable co-localization of both proteins (yellow). The images are the stacks of twelve scans. Scale bar, 20 μm. B. The same area shown on the image of 1-μm-thick scan. The framed areas were enlarged and overlaid to reveal co-localization (right). Numerous MAP-2 positive dendritic profiles contain small, mostly perimembranous, BDNF accumulations. Scale bar, 20 μm. Inset: the arrows point to very small perisomatic BDNF immunofluorescent deposits which do not label for MAP-2.

Most of the BDNF IR elements were dendrites, as indicated by double labeling of BDNF and a dendritic marker MAP-2 (Figure 11). BDNF IR accumulations found in the proximity of large neurons of lamina IX were also identified as the dendritic structures (Figure 11). Many of the BDNF labeled puncta and profiles of various shapes and sizes identified on single optical slices were merged in larger MAP-2 immunolabeled profiles. These characteristics pointed to the presence of local accumulations of BDNF within dendrites. Additionally, the presence of a subset of BDNF IR accumulations which were MAP-2 negative, suggesting their axonal origin, was found at the surface of large neurons of lamina IX (Figure 11).

### Co-localization of BDNF with synaptophysin

**Figure 12:**
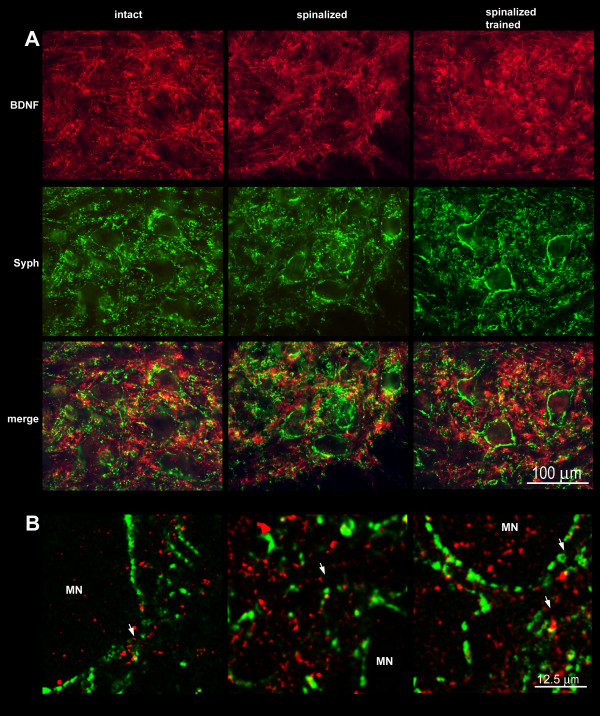
**Co-localization of BDNF (red) and synaptophysin (green) immunofluorescence in double stained cross-sections of lumbar (L3/L4) segments of the spinal cord in the intact (left column), spinal (middle column) and spinal trained (right column) rats**. A. Two-color immunofluorescence photomicrographs demonstrate virtual lack of co-localization of BDNF (red) and synaptophysin (green) in the ventral quadrant of L3/L4 segment of the spinal cord in all experimental groups. Scale bar, 100 μm. B. Representative single confocal scan (1 μm thick), confirming rare co-localization of BDNF and synaptophysin (arrows) in neuropil of motor nuclei. MN- large neurons of lamina IX. Scale bar, 12.5 μm.

**Figure 13:**
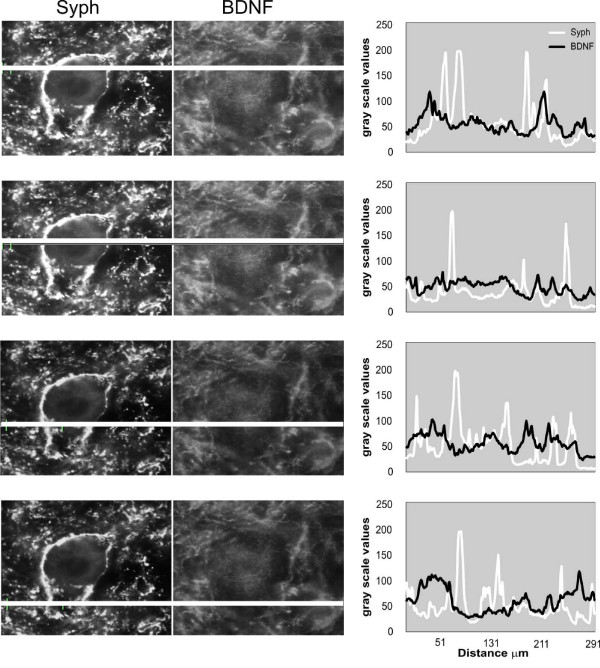
**Distribution of BDNF and synaptophysin immunofluorescence signal across a large neuron of lamina IX**. Left panel: immunofluorescence signal, expressed as gray scale level, was measured in the narrow windows (white horizontal lines). Right panel: peaks of BDNF and synaptophysin signals overlay rarely indicating that these proteins often occupy different cellular compartments.

Although synaptophysin and BDNF immunofluorescence were broadly distributed in the neuropil in the ventral horn, their co-localization was relatively rare. Figure 12 illustrates that the densest accumulation of BDNF IF in the proximity of large neurons of lamina IX does not overlap with that of synaptophysin. This observation is in line with our previous one (see above) on the prevalence of BDNF in dendrites. Irrespective of spinal cord injury and training, strict co-localization of BDNF and synaptophysin was sparse and situated on BDNF IF processes, usually at some distance from the soma of large neurons, probably reflecting loci of pre- and postsynaptic compartments overlap (Figure 12). Analysis of double stained sections under confocal microscopy also confirmed that strict co-localization BDNF IF and synaptophysin IF was very rare (Figure 12). Even in numerous BDNF IR bouton-like accumulations around large neurons of lamina IX, co-localization with synaptophysin was infrequent (Figure 12). Figure 13 exemplifies that the maxima of BDNF and the synaptophysin IF signal seldom overlap in a large neuron of lamina IX and its proximity.

## Discussion

The results presented here show that, six weeks after complete transection of the spinal cord at low thoracic segments, there was an attenuated expression of markers of presynaptic terminals in the ventral quadrants of L3/L4 spinal segments, possibly indicating impoverished innervation of motoneuron pools. Surprisingly, expression of synaptophysin around large neurons of lamina IX and synaptic zinc labeling along fibers of the ventral funiculi, clearly weaker in the spinal than in intact animals, was accompanied by an increased BDNF level in perikarya of large neurons of lamina IX, as well as by a higher number of BDNF IR processes and fibers of altered morphology.

Five-week treadmill locomotor training improved the motor abilities of the spinal rats, confirming beneficial effects of adequate proprio- and exteroceptive, rhythmical stimulation of the hindlimbs and pressure stimulation of the tail [1]. It was accompanied by changes in molecular correlates of compensatory plasticity examined in the neuronal network located caudally to the transection. Namely, an increased number of synaptophysin-labeled nerve terminals upon ventral horn neurons was concomitant with altered levels of zinc-ergic terminals, which tended to increase in the ventral funiculi.

Interestingly, training caused selective enhancement of BDNF IR in perikarya in two out of five subpopulations of cells located in the motor nuclei, whereas expression of BDNF in processes and fibers in the ventral horn tended to be normalized by the training.

### The effect of training of spinal animals on the distribution of markers of presynaptic terminals

#### Synaptophysin

A clear decrease of synaptophysin expression around the large neurons in the motor nuclei of spinal animals and its up-regulation in spinal-trained rats may be interpreted as an enrichment of synapses on the motoneuronal perikarya owing to exercise. Our data do not provide direct evidence on the type of synapses which were enriched. Serotonergic and noradrenergic terminals of the descending fibers retract permanently following complete transection, and this effect excludes them from the pool of boutons under consideration. However, the location and morphology of synaptophysin-positive terminals surrounding perikarya of the large neurons of lamina IX resembled large cholinergic C-boutons [30,31]. A dramatic, sustained decrease of vesicular acetylcholine transporter (VAChT) in the terminals contacting sacrocaudal motoneurons following spinal cord transection at S2 segment, reported by Kitzman [32], indicates that cholinergic projection is vulnerable to the damage and perhaps might be restored by means of locomotor exercise. Indeed, our recent, preliminary data showed that locomotor training of the spinal rats caused an increase of the number of VAChT IR boutons in the triceps surae motoneuron pools in the lumbar segments [33]. These cholinergic terminals may originate from a restricted group of partition interneurons located in the medial part of lamina VII [34]. These interneurons terminate on perikarya and on proximal dendrites of motoneurons and, as documented recently, they potently regulate the excitability of motoneurons during locomotion [34]. Namely, motoneurons are more likely to discharge if excitatory postsynaptic potential (EPSP) amplitude increases and afterhyperpolarization (AHP) decreases; if both occur, stepping of spinal animals was shown to be facilitated [35]. Thus, a decrease of cholinergic input, together with a serotoninergic one, resulting in increased AHP, may contribute to the failure in stepping observed in our experiments.

Other types of terminals contacting motoneuron soma and their proximal dendrites can also be influenced by spinal cord transection and/or by locomotor training. In particular, morphological and biochemical correlates of inhibitory neurotransmission are affected. Both treatments were reported to influence inhibitory GABA and/or glycinergic synapses [36]. Vesicular GABA transporter (VGAT)-expressing terminals, contacting soma and the proximal dendrites of sacro-caudal motoneurons were reported not to increase in number but to increase in size during 12 post-transection weeks [32]. Moreover, a beneficial effect of training on the stepping ability in spinal cats was attributed to attenuation of GABA and glycine synthesis, upregulated after spinal cord transection in the segments caudal to the lesion [37]. Also, in intact rats, four weeks of locomotor training caused a decrease of GABA and glycine levels in the tissue of the L3-L5 segments [38].

The number of glutamatergic terminals expressing vesicular glutamate transporter (VGLUT-2) and found in apposition to the soma and proximal dendrites of motoneurons, markedly increased beginning from the 2^nd ^post-injury week [32]. As a result, the ratio between vesicular transporters of glutamate and GABA (VGLUT-2/VGAT) on the soma of sacrocaudal motoneurons changed dynamically in time after spinal cord transection, finally showing a tendency to spontaneous normalization 12 weeks after injury [32].

All these observations indicate that synapses located on the perikarya and on proximal dendrites of motoneurons, which decreased in number in spinal rats and increased owing to the training, may represent limited phenotypes and may change the pattern of innervation of motoneurons.

The synaptic changes observed in our experiment may stem not only from increasing the number of synapses around motoneurons, but also from enlargement of synaptic territories owing to the training. Enlargement of presynaptic territory of C-terminals has been observed in pathological conditions in transgenic G93A SOD1 mice, during disease progression [39]. In our study we were not able to distinguish between the two mechanisms, as immunofluorescent deposits in spinalized trained animals formed a uniform layer on large areas of the motoneuron membrane. Nevertheless, both mechanisms can lead to changes in synaptic connectivity either through formation of new synapses, or by strengthening the existing ones.

#### Zinc

Widespread distribution of zinc-containing terminals in the spinal cord observed in the intact rats in this study fully confirmed the pattern described previously in rodents with the use of the same method for synaptic zinc visualization [40-42]. It has been suggested that a majority of zinc-containing neurons located in the ventral horn belong to a propriospinal system projecting segmentally or intersegmentally in the ventral gray matter [41]. Here, we reported that after complete transection of the spinal cord, the density of synaptic zinc staining decreased in the ventral funiculi in the segments caudal to the injury site. This observation may partly reflect the changes in both ascending axons and descending proprioceptive fibers originating caudal to spinal transection, which were shown to contribute to the response of motoneurons to stimulation of the ventrolateral funiculi after spinalization [35]. A lack of detectable changes in the dense and convoluted system of zinc-ergic innervation of the ventral horn in the lumbar segments might result from a relatively small, diffused fraction of degenerating fibers bearing zinc-ergic terminals in an extremely rich population of zinc-ergic endings. This observation also indicates that the majority of terminals contacting motoneurons that disappear in the synaptophysin staining are not zinc-ergic ones or that the retracting fibers are replaced by other zinc-ergic terminals. Interestingly, locomotor training of spinal rats produced an increase in density of zinc-ergic terminals in the ventral funiculi, leading to its normalization. This observation strengthens the possibility of reorganization of the neuronal network after post-injury locomotor training that includes axonal sprouting of the ventral propriospinal system and modification of the dendritic tree.

What type of transmission is involved in zinc-ergic network reorganization? In the spinal cord, a majority of zinc-ergic terminals were shown to be GABAergic [41], although synaptic zinc is also present in a subset of glycinergic terminals, as well as in glutamatergic boutons [43,44]. Notably, synaptic zinc released during neurotransmission has direct and indirect actions: it may diminish excitatory neurotransmission, as an inhibitor of NMDA receptors, or act bidirectionally on inhibitory neurotransmission by modulating GABA and Gly receptors, as well as other receptors [45]. A tendency to overall increased inhibitory neurotransmission following spinal cord injury, observed by Tillakaratne and co-authors [32,38] speaks in favor of preferential degeneration of excitatory zinc-ergic nerve terminals. If so, the sprouting of zinc-ergic axons after locomotor training should involve terminals co-releasing glutamate to re-establish the balance between the excitatory and inhibitory inputs.

Formation of inhibitory, preferentially GABAergic synapses [46-49], as well as of glutamatergic synapses [50], was repeatedly reported to be promoted by BDNF, shaping synaptic plasticity. Assuming that a vast majority of synaptic changes in the isolated spinal segments involves such innervation, localization and levels of BDNF immunoreactivity were analyzed to evaluate a relation of BDNF responses to detected synaptic changes.

### The effect of spinal cord transection on BDNF distribution and level

The transection itself caused an overall increase of BDNF IR in neuronal perikarya and in processes and fibers of the ventral horn in the L3/L4 segments. This observation is consistent with that of Zvarova and co-authors [15], who detected, using the technique of ELISA, a higher level of BDNF in the whole tissue homogenate of selected thoraco-lumbo-sacral segments, one and six weeks after complete spinal cord transection at low thoracic level. In that experiment, one week after injury, BDNF level in the L4-5 segments was higher by about 40% than in intact animals and that increase attenuated to 17% by six weeks after transection. Li and co-authors also reported recently that the number of BDNF IR neurons in the ventral horn was increased by over 60% by the end of the first week after complete spinal cord transection at low thoracic segments and that two weeks later it returned to control level [51]. Thus, surprisingly, in spinal rats, which do not demonstrate spontaneous locomotor recovery, it is not an overall BDNF level deficit which seems to be a limiting factor in functional improvement. We assume that it is a limited BDNF availability in the synaptic cleft, which results from disturbances in BDNF synaptic release, and/or altered expression of TrkB receptors, particularly TrkB truncated forms, shown to limit BDNF signaling *in vivo *[52]. Support for this hypothesis stems from the studies that showed an increase of expression of truncated TrkB, detected four weeks after spinal hemisection [53] and seven weeks after contusion of the spinal cord [54]. On the other hand, local synaptic accumulation of BDNF released from overloaded terminals might desensitize TrkB full length (FL) receptors, downregulating neurotrophin signaling, as shown by us in *in vitro *model [55]. In addition, a deficit of zinc ions, which can decrease transactivation of the synaptic TrkB by a neuronal activity-regulated mechanism [56,57], may attenuate TrkB signaling. These disturbances may affect the strengthening of synaptic connections owing to desynchronized firing of the presynaptic and postsynaptic neurons [58], discussed by Petruska et al. [35].

It is worth consideration that the effect of other types of spinal cord injury on BDNF mRNA and protein levels in the lumbar spinal cord of the rat was different from that after complete transection [14,59,13,61]. Thus, the extent of spinal cord injury substantially influences the expression of BDNF mRNA and protein in the region caudal to the injury site, suggesting the role of descending pathways in this regulation. However, Garraway and Mendell [62] attributed these physiological differences to cellular changes characteristic of these two types of injury rather than to an interruption of descending inputs, as they have been observed both caudally and rostrally to the lesion site.

An hypothesis of increased excitability of the central pattern generator (CPG) in chronic spinal animals might be useful to explain up-regulation of BDNF caudally to the lesion site [63]. An increase of the BDNF level in the ventral horn neurons, which was sustained several weeks after transection, may be indicative of the compensatory response of the regions deprived of the descending innervation but still receiving peripheral inputs. First, since a majority of ventral horn neurons can synthesize BDNF [8,64], its higher level reflects the recovery potential of these neurons. Second, as BDNF expression is activity-dependent, it may also be indicative of an increased drive to ventral horn neurons.

A cutaneous input, indirectly activating motoneurons via interneurons, could be a good candidate responsible for an increased drive to spinal neurons [65-67]. The motoneurons innervating hindlimb muscles were frequently driven by cutaneous input from the dorsal aspect of the paraplegic hindlimbs, which could keep BDNF up-regulated. If BDNF release processes were undisturbed or enhanced, we could expect that BDNF would elevate presynaptic transmitter release, as reviewed by Poo [68]. Our observation that, in the spinal animals, the enhanced levels of BDNF appeared in shorter and thinner fibers and processes than in the intact and spinal trained ones, may result from their shrinkage or generation of sprouts after spinalization and be indicative of altered presynaptic mechanisms. Also, it may explain an impoverishment in neural networks, as indicated by a reduced synaptophysin expression around the large neurons in the motor nuclei, together with a decreased number of zinc grains along the processes and fibers in the ventral funiculi. Postsynaptic responses to altered levels of neurotransmitters may be further modified by BDNF. BDNF may differentially affect an effectiveness of neurotransmission, depending on the type of synapse and postsynaptic cell, as shown for GABAergic synapses on GABAergic neurons, where BDNF decreased the efficacy of inhibitory transmission [69].

### The effect of training in spinal animals on BDNF distribution and level

Surprisingly, training did not influence the overall levels of BDNF immunoreactivity either in perikarya or in processes and fibers of the ventral quadrant, compared with those of the spinal non-trained animals. However, two subsets of the ventral horn cells tended to respond with higher BDNF levels to the locomotor training. One of them, with soma size ranging between 100 and 400 μm^2^, represented a mixed population, which could consist of, for example, γ-motoneurons [70], interneurons, or even glial cells. The other cells, with the perikarya exceeding 1000 μm^2 ^presumably corresponded to α-motoneurons [70]. Our previous studies showed that locomotor training caused up-regulation of BDNF mRNA in practically all types of cells, differentiated by size, in the intact spinal cord [8]. A question arises about the physiological meaning of an increased, selective expression of BDNF in some cells of the ventral horn in trained spinal rats.

A possible explanation may be found in the experiments showing that the amplitude of monosynaptic Hoffmann reflex in the soleus muscle increases by a factor of two when preceded by tactile stimulation of the tail in the awake, intact rat [71]. It is thus feasible that, in spinal animals, treadmill walking, which produces adequate activation of proprioceptive input from the plantar aspect of the feet when supplemented with the pressure of the tail, may favor activation of extensor motoneurons [65,72]. This effect would explain the selective up-regulation of BDNF in the neurons of motor nuclei in spinal trained animals. Indeed, our recent experiment showing an increase in the number of cholinergic boutons apposing the extensor but not the flexor motoneurons after training, speaks in favor of this possibility [33].

Training caused changes in the distribution of BDNF in the processes and fibers, leading to its normalization. More numerous, but shorter and thinner BDNF IR processes and fibers, were detected in the spinal animals, versus longer and thicker ones in the spinal trained rats. This observation, when associated with selective up-regulation of BDNF in some cells owing to the training, might point to their dendritic origin. Indeed, as identified with double immunolabeling, many of the BDNF IR profiles were dendrites. They can represent a dendritic tree of spinal motoneurons, enriched owing to locomotor training. Such enrichment was described by Gazula and co-workers after five days of physical training in spinal animals [73]. In contrast to the observations by Ying and co-workers [14] showing that BDNF protein levels were increased in proportion to the amount of voluntary exercise performed after hemisection of the spinal cord, we did not find such a relationship.

Taken together, our data indicate that locomotor training caused redistribution of BDNF to selected groups of spinal cells rather than influenced the general level of BDNF caudally to the site of transection. This observation may indicate that selective up-regulation of BDNF promotes rewiring of the spinal neuronal network activated by kinesthetic stimuli associated with locomotion and by pressure stimulation of the tail. Moreover, it indicates that, in functional reorganization, not only the availability of a releasable pool of BDNF, but also its proper patterning, is crucial.

### Co-localization of BDNF with pre- and postsynaptic markers

The number of co-localized profiles of synaptophysin and BDNF in the ventral horn was small. This result was surprising in view of the recent data pointing to a role of BDNF in the regulation of the expression of presynaptic proteins involved in synaptic vesicle fusion and in synapse formation [68]. Three days of intensive, voluntary locomotor exercise were reported to lead to an increase of synapsin I and synaptophysin proteins level in the hippocampus, which was abolished when BDNF signaling via TrkB receptor was blocked [50,74]. It is worth stressing that Pozzo-Miller and colleagues observed a reduction of synaptophysin in hippocampal synaptosomes in BDNF knockout mice [21].

An unexpectedly small number of co-localized profiles of synaptophysin and BDNF in the ventral horn may derive from the fact that both proteins, present in synapses, occupy different subcellular compartments. Synaptophysin is a component of all synaptic vesicles, whereas BDNF is present only in a subpopulation of endosomes. As a result, relatively small deposits of BDNF IF (see Figure 12) may be overshadowed by intensive synaptophysin IF profiles of numerous synaptic vesicles, which build large spots of signal in terminals and their clusters.

We also questioned the identity of the BDNF IR bouton-like accumulations around large neurons of lamina IX. Rare co-localization of synaptophysin and BDNF IR in these structures suggested that the latter do not represent BDNF-driven active terminals. Indeed, some of these accumulations corresponded to dendritic structures, as revealed by strong MAP-2 staining with double-labeled (BDNF/MAP-2) profiles localized in the proximity of large neurons. A deficiency in synaptophysin IR around large neurons of lamina IX after spinalization and its high increase in terminals owing to training were surprising in view of similarly strong expression of BDNF in this region in both groups. This discrepancy may partly result from the fact that, as shown by us, some of the reappearing terminals in trained rats do not carry BDNF, albeit it does not exclude their dependence on BDNF.

## Conclusion

Our data indicate that in the spinal rats, which do not demonstrate spontaneous locomotor recovery, it is not an overall BDNF level deficit which seems to be a limiting factor in functional improvement. Rather, a restricted BDNF availability in the synaptic cleft and/or altered expression of TrkB receptors, particularly TrkB truncated forms, may attenuate signaling in target motoneurons and limit functional recovery.

The training-associated higher accumulation of BDNF in two distinct subpopulations of cells in the motor nuclei as well as in longer and thicker processes, together with increased markers of synaptic boutons terminating upon large neurons in the areas occupied by motoneuronal processes reflect a substantial change of innervation targeting spinal motoneurons, which are tuned up by locomotor activity reinforced with the tail stimulation, resulting in motor improvement.

## Authors' contributions

MM participated in the design of the study, carried out behavioral experiments, surgery and immunocytochemical experiments concerning BDNF expression in the spinal cord, performed data analysis, partly wrote a draft of the article, prepared illustrations, and edited their final version. DN participated in the design of the study, carried out immunocytochemical experiments concerning synaptophysin expression in the spinal cord, performed data analysis, prepared illustrations, and wrote part of the draft of the article. AC participated in the design of the study, carried out the experiments concerning zinc expression in the spinal cord, performed data analysis, prepared illustrations, and wrote part of the draft of the article. DS carried out behavioral studies and participated in the design of immunocytochemical experiments concerning BDNF expression in the spinal cord. MS conceived and co-designed the study, supervised and performed immunocytochemical experiments concerning BDNF expression in the spinal cord, and helped to write a draft of the article. JS-K participated in the design and helped in coordination of the study, and wrote part of the draft of the article. JC-B conceived and co-designed the study, supervised and performed surgery and behavioral study, coordinated the study, and drafted the article. All authors read and approved the final manuscript.
